# Reporting of Drug-Induced Myopathies Associated with the Combination of Statins and Daptomycin: A Disproportionality Analysis Using the US Food and Drug Administration Adverse Event Reporting System

**DOI:** 10.3390/jcm12103548

**Published:** 2023-05-18

**Authors:** Chunyan Wei, Wanhong Yin, Zhiyao He, Bin Wu

**Affiliations:** 1 Department of Pharmacy, West China Hospital, Sichuan University, Chengdu 610041, China; cyw_hx611@wchscu.cn (C.W.); zhiyaohe@scu.edu.cn (Z.H.); 2 Department of Critical Care Medicine, West China Hospital, Sichuan University, Chengdu 610041, China; yinwanhong@wchscu.cn; 3 West China School of Clinical Medical College, Sichuan University, Chengdu 610041, China; 4 West China School of Pharmacy, Sichuan University, Chengdu 610041, China

**Keywords:** daptomycin, statins, myopathy, rhabdomyolysis, FAERS database

## Abstract

Background: Myopathy is one of the most common adverse reactions of daptomycin and statins. We aimed to evaluate the muscular toxicity of the combination therapy of daptomycin and statins in a large pharmacovigilance database. Methods: This was a retrospective disproportionality analysis based on real-world data. All cases reported between the first quarter of 2004 and the fourth quarter of 2022 where daptomycin and statins were reported were gathered from the US Food and Drug Administration Adverse Event Reporting System (FAERS) database. Disproportionality analyses were conducted by estimating the proportional reporting ratios (PRRs), reporting odds ratio (ROR), and information component (IC). Results: A total of 971,861 eligible cases were collected from the FAERS database. Data analysis showed that rosuvastatin (ROR: 124.39, 95% CI: 87.35–178.47), atorvastatin (ROR: 68.53, 95% CI: 51.93–90.43), and simvastatin (ROR: 94.83, 95% CI: 71.12–126.46) combined with daptomycin increased the reporting frequency of myopathy. Moreover, myopathy was reported more frequently with the 3-drug combination (ROR: 598.01, 95% CI: 231.81–1542.71). For rhabdomyolysis, the frequency of reports also increased when daptomycin was combined with rosuvastatin (ROR: 156.34, 95% CI: 96.21–254.05), simvastatin (ROR: 72.65, 95% CI: 47.36–111.44), and atorvastatin (ROR: 66.31, 95% CI: 44.06–99.81). Conclusions: The combination of daptomycin and statins increased the association of myopathy and rhabdomyolysis, especially with rosuvastatin, simvastatin, and atorvastatin.

## 1. Introduction

Drug-induced myopathies (DIMs) are one of the most common causes of myopathy. The manifestations of DIMs range from mild myalgia to chronic disease with myasthenia gravis and rhabdomyolysis with acute renal failure, which may lead to death in severe cases [1,2]. More than 150 drugs can cause DIM, among which the most famous include statins and long-term high-dose glucocorticoids [3,4,5,6]. The pathogenesis of DIM has not been clearly defined, and it is generally believed that the mechanisms include direct myotoxicity (e.g., statins and chloroquine), immune-mediated myopathy (e.g., checkpoint inhibitors and statins), and indirect muscle damage (e.g., diuretic-associated hypokalemia) [7,8]. In some settings, multiple mechanisms may combine to produce muscle damage, and drug combinations may enhance myopathy. As an example, erythromycin, diltiazem, and azole antifungals increase the muscle toxicity of statins due to the increasing serum concentration of statins by the competition for cytochrome P450 metabolism in the liver [9]. DIMs may also develop because of the additive myotoxic effects of multiple drugs. For example, amiodarone may result in vacuolar changes in muscle histology when used in combination with statins or colchicine [10,11].

Daptomycin is a cyclic lipopeptide antimicrobial, which can cause calcium-dependent depolarization of the cell membrane of Gram-positive bacteria, including strains of Staphylococcus aureus and Enterococcus spp. both susceptible and resistant to classic antimicrobials, and has become a widely used anti-positive bacteria drug in the clinic [12,13]. The US Food and Drug Administration (FDA) and European Medicines Agency (EMA) have approved daptomycin for treatment in complex skin and soft tissue infections, as well as aureus bacteremia with complex skin and soft tissue infections or right-sided infective endocarditis. The most commonly reported adverse effects of daptomycin were myopathy and eosinophilic pneumonia [14]. However, daptomycin-induced myopathy has been described in about 2–14% of patients receiving daptomycin [15], and rhabdomyolysis was as high as 5% [16,17,18,19]. The factors conferring an increased risk for daptomycin-induced myopathy or rhabdomyolysis may be dose-related [20]. Daptomycin is recommended to be dosed based on total body weight, and patients with baseline renal impairment and obesity may have increased daptomycin exposure [20,21,22,23].

As previously mentioned, the combination of multiple muscular-toxic drugs may increase the risk of DIM. At present, there are many contradictions in research results. In a case–control study, patients treated with daptomycin who developed myopathy were matched with controls who did not develop myopathy, and statin co-administration was an independent risk factor for myopathy (odds ratio: 2.60; *p* = 0.03) and rhabdomyolysis (odds ratio: 4.67; *p* = 0.03) [15]. However, a number of retrospective studies have observed no increased risk of DIM with statin co-administration [24,25,26,27], even in high-dose daptomycin therapy (≥10 mg/kg·day) [27]. Both statins and daptomycin are widely used in the clinic. Whether they increase DIM in co-administration is a subject worthy of attention, in particular an increase in the risk of rhabdomyolysis. Therefore, the aim of our study was to explore whether the combination of statins and daptomycin would increase the reporting rate of DIM based on real-world data from the FDA Adverse Event Reporting System (FAERS) database. It is expected to provide a reference for the security of drug usage in clinical practice and provide direction for pharmaceutical care by clinicians and pharmacists.

## 2. Materials and Methods

### 2.1. Data Source, Extraction, and Processing

This was a retrospective, observational pharmacovigilance study designed to analyze the DIM events associated with daptomycin and statin co-administration. The data came from reports in the FAERS database from the first quarter of 2004 to the fourth quarter of 2022. The FAERS database is a self-reporting database that collects adverse drug event reports with a large amount of information and used for post-marketing drug safety surveillance. Data sources of FAERS are public, and the collected data include patient demographic information, drug information, drug combination, and patient outcome information. A report may have one or more adverse drug events and are reported with anonymous patient information. Therefore, no ethical approval was required, and informed consent could not be obtained.

The classification and standardization of adverse reactions in the FAERS database refer to the Medical Dictionary for Regulatory Activities (Med DRA), and each report is coded using preferred terms (PTs) from the MedDRA terminology. One PT can correspond to one or more High-Level Group Terms (HLGTs), High-Level Terms (HLTs), and System Organ Class (SOC) levels in MedDRA. Different PTs can also be collected to define a specific adverse reaction through standardized MedDRA queries (SMQs) [28].

This study relied on definitions used by MedDRA. The FAERS consists of seven data modules, including patient demographic and administrative information (DEMO), drug information (DRUG), patient outcomes (OUTC), adverse events (REAC), report sources (RPSR), indications for drug administration (INDI), and therapy start states and end dates for reported drugs (THER). A clean, drug-mapped, de-duplicated version of the FAERS data was extracted according to the FDA recommendations [29]. If the CASEIDs (a number used to identify a FAERS case) were the same, the latest FDA_DT (date FDA received the case) was selected. If the CASEID and FDA_DT were the same, the higher PRIMARYID (a unique number for identifying a FAERS report) was selected. Then, we used the MedEx 1.3.8 software to standardize different names of the same drug into the “generic name” [30].

The REAC module and INDI module were both coded by MedDRA preferred terms [31]. We collected all the cases of daptomycin or statins as primary suspected (PS) and non-primary suspected drugs reported in FAERS database. A primary suspected drug refers to a reporter listing a specific drug as the main suspect drug when reporting adverse drug reactions. We divided the reports into a DIM group and non-DIM group. The DIM cases were defined according to the High-Level Group Term (HLGT, coded by MedDRA, narrow: 20000002), incorporating 32 PTs as shown in Supplementary Table S1. We attempted to identify the daptomycin and 8 statins according to the WHO Anatomical Therapeutic Chemical (ATC) classification from the local FAERS database. The ATC codes are shown in Supplementary Table S2. After indication identification, we eliminated cases with DIM cases reported in the INDI module.

### 2.2. Statistical Analysis

We managed the FAERS dataset in local use through Microsoft SQL Server 2017 software. The characteristics of DIM cases and non-DIM cases with target drugs were collected, including age, sex, report year, report country, identity of reporter (health professionals or non-health professionals). Algorithms of the reporting odds ratio (ROR) and information component (IC) were used to detect the association between DIM events and target drugs [32]. EXCEL software (version 2304 build 16.0.16327.20200) was used to calculate the value of ROR and IC, including the 95% confidence interval (95% CI) of them. For ROR, the significant association was detected when the case number was ≥3 and the lower limit of the 95% CI was >1. For the IC method, if IC > 0 and the lower limit of 95% CI was >0, the signals were considered significant [33]. The ROR value was used as the primary assay, and the IC value was used as the confirmation method. The DIM events were considered to be associated with the target drug only when both the ROR and the IC methods met their threshold. The calculation methods of ROR, IC, and 95% CI are shown in Supplemental Table S3.

## 3. Results

### 3.1. DIM Event Identification in the FAERS Database

**Figure 1:**
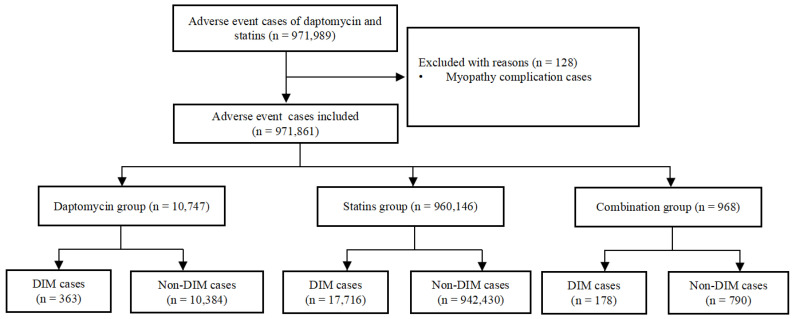
Flowchart of identifying adverse event cases of daptomycin and statins from the FAERS database. DIM: drug-induced myopathies.

We identified a total of 971,989 adverse event cases with daptomycin and statins in the FAERS database, of which 128 cases with a complication of myopathy were excluded. Finally, 971,861 cases were included in the disproportionality analysis; 18,257 (1.88%) cases were assigned to the DIM group and 953,604 (98.12%) to the non-DIM group. The number of adverse reactions reported with statins was significantly higher than with daptomycin (10,747 cases in the daptomycin group; 960,146 cases in the statins group). In addition, a total of 968 cases involved statin and daptomycin co-administration. The combination group had the highest proportion of DIM events, and the proportion of DIM events in the 3 groups was 3.38%, 1.85%, and 18.39%. The details of the case identification are shown in Figure 1.

### 3.2. Demographic Characteristics

The characteristics of the events are described in Table 1. In the DIM group, the proportion of males was higher than females (55.67% vs. 35.58%), which was not the case in the non-DIM group (45.62% vs. 48.51%). The reported rate of myopathy appeared to be higher in males. Adverse events were more frequently reported in patients older than 65 years in both groups (DIM group: 48.66%; non-DIM group: 44.11%). The number of DIM events reported in patients younger than 18 years was extremely low (*n* = 55, 0.30%). Regarding the identity of the reporter, most of the cases were reported by health professionals, especially in the DIM group (82.86%). More than 70% of the reports came from Europe and North America. Europe reported the highest number of DIM events (42.09%), with the highest number of non-DIM events coming from North America (64.31%). From 2004 to 2018, the average annual number of reports did not change significantly. However, there was a slight increase in the number of reports for 2019–2022.

### 3.3. DIM Signal Detection in Daptomycin and Statins Based on the Primary Suspect

**Figure 2:**
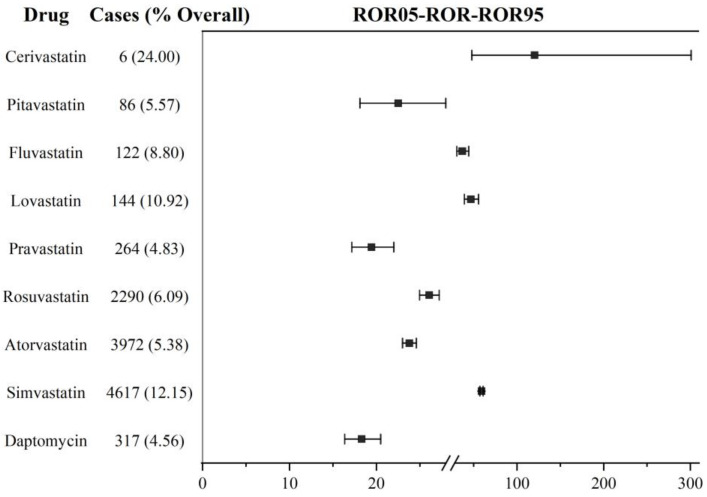
DIM signal detection in daptomycin and statins based on the primary suspect. ROR: reporting odds ratio; the squares in the figure represent the ROR value, and the line segment represents the 95% confidence interval of ROR.

Firstly, we analyzed DIM event reporting frequency for the non-combination of daptomycin and statins based on the primary suspect drug. With the exception of cerivastatin, which was delisted in the US in 2001, daptomycin and other statins showed significant DIM signals. The ROR values in descending order were simvastatin (ROR: 59.01, 95% CI: 57.13–60.95; IC: 5.53, 95% CI: 5.41–5.63), lovastatin (ROR: 46.78, 95% CI: 39.34–55.64, IC: 5.38, 95% CI: 4.45–5.59), fluvastatin (ROR: 36.83, 95% CI: 30.57–44.36; IC: 5.07, 95% CI: 4.12–5.34), rosuvastatin (ROR: 26.06, 95% CI: 24.96–27.22; IC: 4.54, 95% CI: 4.38–4.67), atorvastatin (ROR: 23.78, 95% CI: 22.99–24.59; IC: 4.36, 95% CI: 4.24–4.46), pitavastatin (ROR: 22.50, 95% CI: 18.10–27.97; IC: 4.41 95% CI: 3.39–4.83), pravastatin (ROR: 19.44, 95% CI: 17.17–22.00; IC: 4.20, 95% CI: 3.70–4.52), daptomycin (ROR: 18.30, 95% CI: 16.34–20.49; IC: 4.12, 95% CI: 3.67–4.42). The lower limit of the 95% CI for all the ROR values showed above was >1, and of IC values was >0. Cerivastatin had the highest ROR value (120.16, 95% CI: 47.99–300.88) compared with the other drugs, but the lower limit of the 95% CI for IC was lower than 0 (IC: 6.52, 95% CI: (−0.06)–5.48). That might relate to its early delisting. In the analysis based on the primary suspect drug, the association between daptomycin and myopathy was weaker than all the statins. In terms of the number of reports, simvastatin (*n* = 4617), atorvastatin (*n* = 3972), and rosuvastatin (*n* = 2290) had the largest numbers of DIM reported events and were 10 times higher than other drugs. See Figure 2 for details.

### 3.4. DIM Signal Detection in Daptomycin and Statin Co-Administration

**Figure 3:**
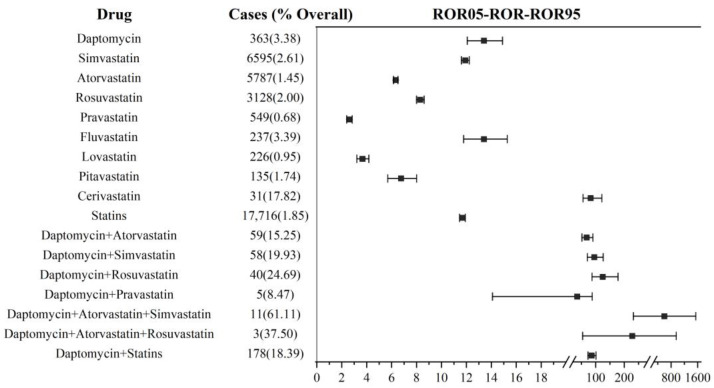
DIM signal detection in daptomycin and statin co-administration. ROR: reporting odds ratio; the squares in the figure represent the ROR values, and the line segment represents the 95% confidence interval of ROR.

As shown in Figure 3, the DIM event reporting frequency analysis was executed based on all reports, which included the primary suspect drug and non-primary suspect drug. In statins, cerivastatin again had the highest ROR (82.54, 95% CI: 55.97–121.71; IC: 6.09, 95% CI: 3.20–5.71), and fluvastatin came in second (ROR: 13.41, 95% CI: 11.78–15.27; IC: 3.69, 95% CI: 3.19–4.05). Pravastatin had the lowest reporting frequency (ROR: 2.61, 95% CI: 2.40–2.84; IC: 1.37, 95% CI: 1.08–1.64). The reporting frequency of daptomycin (ROR: 13.41, 95% CI: 12.07–14.89; IC: 3.69, 95% CI: 3.29–3.99) was similar to that of fluvastatin. Overall, statins were reported less frequently (ROR: 11.68, 95% CI: 11.45–11.91; IC: 2.82, 95% CI: 2.76–2.87) than daptomycin, which was different from the analysis results based on the primary suspect drug.

For co-administration, a total of 6 combination treatment possibilities were identified; 4 of these regimens involved 2-drug combinations, and 2 regimens involved 3-drug combinations including 178 cases. Among them, the lower limit of the 95% CI for the IC values of daptomycin+ pravastatin (IC: 5.01, 95% CI: (−0.41)–5.16) and daptomycin+ rosuvastatin+ atorvastatin (IC: 7.16, 95% CI: (−1.95)–5.88) was less than 0, and the significance needed to be further confirmed. In the end, we defined four meaningful co-administration regimens, described below.

In all four combination regimens, the DIM events reported an increase in frequency compared with their non-combination treatment. Daptomycin combined with rosuvastatin (ROR: 124.39, 95% CI: 87.35–178.47; IC: 6.56, 95% CI: 3.71–5.98) was higher than the combination of simvastatin (ROR: 94.83, 95% CI: 71.12–126.46; IC: 6.25, 95% CI: 4.13–5.99) and atorvastatin (ROR: 68.53, 95% CI: 51.93–90.43; IC: 5.86, 95% CI: 3.99–5.80). The ROR value (598.01, 95% CI: 231.81–1542.71; IC: 7.86, 95% CI: 1.11–5.92) of 3-drug co-administration (daptomycin+ simvastatin+ atorvastatin) was higher than that of 2-drug co-administration. In general, the total DIM reporting frequency (ROR: 86.08, 95% CI: 73.14–101.31; IC: 6.13, 95% CI: 5.13–6.19) by daptomycin combined with statins was higher than when they were used separately. For other statins not shown in Figure 3, there were less than three reported cases of rhabdomyolysis in combination therapy, and no ROR value was calculated.

### 3.5. Rhabdomyolysis in Daptomycin and Statin Co-Administration

**Figure 4:**
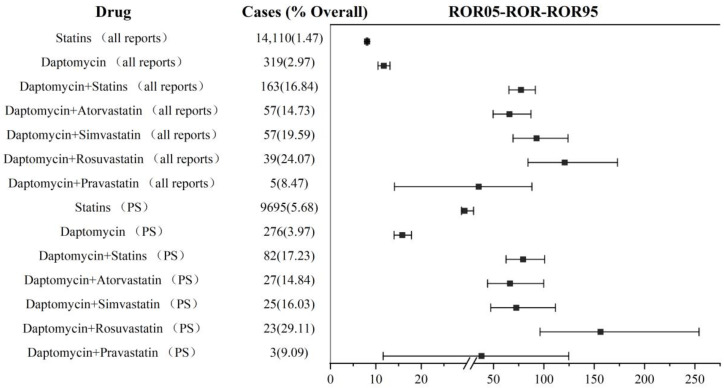
Signal detection in daptomycin and statin co-administration. ROR: reporting odds ratio; PS: primary suspected; the squares in the figure represent the ROR value, and the line segment represents the 95% confidence interval of ROR.

We separately calculated the reporting frequency of rhabdomyolysis (PTs: 10039020). The analysis results based on the primary suspect drug (ROR: 79.33, 95% CI: 62.52–100.66; IC: 6.04, 95% CI: 4.43–5.98) and all reports (including primary suspect drug and non-primary suspect drug) (ROR: 77.33, 95% CI: 65.33–91.54; IC: 6.01, 95% CI: 4.98–6.08) showed that the co-administration of daptomycin and statins may increase the association with rhabdomyolysis more than non-combination therapy. Major increases in reporting frequency were also due to the combination of rosuvastatin (PS: ROR: 156.34, 96.21–254.05; IC: 6.80, 95% CI: 2.79–5.83. All reports: ROR: 120.74, 95%CI: 84.21–173.12; IC: 6.52, 95% CI: 3.66–5.96), simvastatin (PS: ROR: 72.65, 95% CI: 47.36–111.44; IC: 5.93, 95% CI: 2.82–5.58. All reports: ROR: 92.80, 95% CI: 69.46–123.98; IC: 6.22, 95% CI: 4.10–5.97), and atorvastatin (PS: ROR: 66.31, 95% CI: 44.06–99.81; IC: 5.82, 95% CI: 2.92–5.56. All reports: ROR: 65.80, 95% CI: 49.67–87.18; IC: 5.81, 95% CI: 3.93–5.76). Whether the co-administration of daptomycin and pravastatin (PS: ROR: 38.05, 95% CI: 11.61–124.68; IC: 5.12, 95% CI: (−1.56)–5.32. All reports: ROR: 35.23, 95% CI: 14.09–88.08; IC: 5.01, 95% CI: (−0.41)–5.16) increased rhabdomyolysis needed to be further verified, due to the lower limit of the 95% CI for IC being <0. The details are shown in Figure 4. For other statins not shown in Figure 4, there were less than three reported cases of rhabdomyolysis in combination therapy, and no ROR value was calculated.

## 4. Discussion

Myopathy is one of the most concerning adverse effects of statins, and it is the most common reason for non-adherence and therapy discontinuation [34,35,36]. The incidence of myopathy and rhabdomyolysis was high in patients treated with daptomycin [3,37]. Since both statins and daptomycin are widely used in clinical practice, whether their co-administration leads to an increased risk of myopathy has been considered by researchers [27,38]. In previous studies, researchers have conducted disproportionality analysis of the correlation between the combination of daptomycin and statins and DIM occurrence [39,40]. However, there were some limitations in each of these studies. In Tomoyukiv’s research [40], the data source was the Japanese Adverse Event Report database, and the outcomes of muscle toxicity were only defined by the elevation of creatine phosphokinase (CPK) levels, myopathy, and/or rhabdomyolysis. In addition, this study involved a small sample size (*n* = 250). Masayuki [39] conducted a meta-analysis to explore whether statins increased the incidence of daptomycin-associated myopathy. At the same time, a disproportionality analysis was performed based on the FAERS database to further confirm the results of the meta-analysis. Only 11 PTs were considered for inclusion in this study, and only ROR values were calculated as the basis for judgement. However, all of these studies showed positive results. To the best of our knowledge, our study included the largest number of PTs (*n* = 32), the largest sample size (*n* = 971,861), and the most recent data (retrieved until the fourth quarter of 2022). Furthermore, we used two data analysis methods (ROR and IC) to confirm the reliability of the results.

We found that the DIM group reported a higher proportion of males. This contradicted previous research findings. In an observational study, myopathy was reported frequently in women [41]. The risk ratio for women was 1.52 as compared with men, and 80% of the women compared with 43% of the men reported that the muscular symptoms affected their daily activities [41]. We did not confirm the reason for this discrepancy. It might be due to reporting bias or differences among ethnic groups. In the DIM group, reports from non-health professionals accounted for 9.36%, much less than in the non-DIM group (40.79%). We speculated that there might be a lack of understanding of DIM among non-health professionals because it requires a certain medical basis to make a judgment. Perhaps in the follow-up work the popularization of DIM can be strengthened.

It was worth noting that all types of statins were significantly associated with DIM, even those with a lower risk of myopathy confirmed by previous studies [42,43,44], such as fluvastatin, pravastatin, and pitavastatin. The muscular toxicity of these drugs in clinical treatment was still noteworthy. The combination of daptomycin and statins increased the reporting frequency of DIM, and the reporting frequency of the three-drug co-administration was higher than that of the two-drug co-administration. As far as we know, the combined use of two statins was uncommon in clinical practice. The co-administration of two statins might be present in patients with statin switching. Statin switching occurs mostly because of statin intolerance, with the most common cause being myalgia [45]. This suggests that, if the patient is intolerant to statin because of myopathy, the association with myopathy may be higher when using daptomycin in combination. In the two-drug combination regimen, only cases of rosuvastatin, atorvastatin, and simvastatin combined with daptomycin were found. This might be related to the wider clinical use of these three statins [46,47], and we could not rule out the increased correlation of DIM with a combination of other statins and daptomycin. We recommend that all types of statins should be more closely monitored for creatine kinase and myalgia symptoms when in co-administration with daptomycin. If possible, consider stopping statins first and restarting treatment after the completion of the daptomycin course. A randomized controlled trial of 17,802 adults showed that rosuvastatin at 20 mg/day did not cause a significant increase in myopathy [48]. However, according to our results, rosuvastatin combined with daptomycin had the highest correlation with DIM compared with the other statins. This might be related to the widespread use of rosuvastatin in clinical practice and the large increase in the patient base.

Rhabdomyolysis is the most severe condition of DIM; the cases are rarer but have a significant mortality [49]. It is caused by the breakdown and necrosis of muscle tissue and release of intracellular content into the blood stream. The most common inducements of rhabdomyolysis are trauma and drugs. The most feared complication of rhabdomyolysis is development of acute kidney injury (AKI) [50]. It is estimated that 10–40% of rhabdomyolysis cases are complicated by AKI, and the patients who develop AKI have an increased mortality rate as high as 80% [51]. The risk factors for rhabdomyolysis include age (older than 50 years), initial creatinine (greater than 1.4 mg/dL), initial calcium (<7.5 mg/dL), initial creatine kinase (>40,000 U/L), etc. [50]. Perhaps the most frequent cause of drug-induced rhabdomyolysis is the administration of statins. Statins in combination with daptomycin might also increase the reporting rate of rhabdomyolysis, especially with rosuvastatin, atorvastatin, and simvastatin. Rosuvastatin and daptomycin co-administration was most frequently reported. Great care needs to be taken with rhabdomyolysis when statins are used in combination with daptomycin.

A number of pathophysiological hypotheses have been proposed to explain the muscular toxicity caused by statins, but none of them have been unequivocally proved. Possible mechanisms include gene regulation and polymorphisms, mitochondrial disfunction, and a decrease in protein prenylation and coenzyme Q10 [52]. The mediated effects of statin inhibition on HMG-CoA reductase perturb the mevalonate pathway, and this perturbation has been linked to possible statin-negative effects on muscle. The impairment of these pathways could alter energy metabolism and determine the formation of lipid-filled vacuoles and fiber atrophy [53]. High lipophilicity makes it easier for statins to penetrate membranes, resulting in a wide distribution in different tissues, leading to more favorable cardiovascular outcomes. However, lipophilic statins are mainly metabolized by the cytochrome P450 (CYP 450) enzyme, and the muscle damage risk is vulnerable to drug–drug interactions [52]. Pravastatin and fluvastatin were the least likely to cause muscle cell damage, which is at least partly related to the fact that they are not metabolized by the CYP 3A4 pathway [54]. Their strong incorporation into the muscle may possibly be associated with the muscle injury of daptomycin [55]. Daptomycin has little effect on CYP 450 enzyme-related metabolism. The mechanism of increasing DIM in combination with statins has not been elucidated.

There are some important limitations inherent to the use of the FAERS database. First, the FAERS database is a spontaneous reporting system; therefore, the potential reporting bias is hard to avoid. For example, health professionals pay more attention to severe and new adverse reactions and may choose not to report minor adverse events. This may also explain why more myopathy events were reported by health professionals. Second, causality cannot be inferred or determined as the patient treatment information is often incomplete, including patient history and the timing of the reported medication use. We were not able to identify other factors that might have influenced the results. Finally, the mechanism of the increased association of adverse reactions cannot be described in our study. In subsequent research, we expect more high-quality studies to prove the causality of statins combined with daptomycin on the incidence of DIM, such as randomized controlled trials or case–control studies, and to provide a more detailed explanation of its pathogenesis.

## 5. Conclusions

The combination of daptomycin and statins increased the association of myopathy and rhabdomyolysis, especially with rosuvastatin, simvastatin, and atorvastatin. Rosuvastatin was associated with the highest reporting frequency, including rhabdomyolysis. There is a higher rate when rhabdomyolysis occurs; the enhanced monitoring of creatine kinase and myalgia symptoms is recommended in statin and daptomycin co-administration patients in clinical practice. In order to minimize adverse drug reaction, when two types of drugs need to be used in combination, consider stopping statins first and restarting treatment after the completion of the daptomycin course, or consider switching to other types of lipid-regulating drugs with less muscular toxicity.

## Figures and Tables

**Table 1:** Demographic characteristics of cases.

	DIM Group		Non-DIM Group		Total
	Number (*n*)	Proportion (%)	Number (*n*)	Proportion (%)	Number (*n*)
**Gender**					
Female	6495	35.58	462,610	48.51	469,105
Male	10,164	55.67	435,023	45.62	445,187
Unknown	1598	8.75	55,971	5.87	57,569
**Age (year)**					
<18	55	0.30	1532	0.16	1587
18–65	6060	33.19	288,139	30.22	294,199
≥65	8883	48.66	420,630	44.11	429,513
Unknown	3259	17.85	243,303	25.51	246,562
**Identity of reporter**					
Health professional	15,128	82.86	488,562	51.23	503,690
Non-health professional	1709	9.36	388,956	40.79	390,665
Unknown	1420	7.78	76,086	7.98	77,506
Europe	7684	42.09	214,147	22.46	221,831
North America	6640	36.37	613,283	64.31	619,923
Asian	1497	8.20	38,068	3.99	39,565
Oceania	509	2.79	8806	0.92	9315
South America	77	0.42	18,802	1.97	18,879
Africa	33	0.18	2712	0.28	2745
Unknown	1817	9.95	57,786	6.06	59,603
**Report year**					
2004–2008	4470	24.48	101,399	10.64	105,869
2009–2013	4665	25.55	211,535	22.18	216,200
2014–2018	4195	22.98	336,073	35.24	340,268
2019–2022	4927	26.99	304,597	31.94	309,524

## Data Availability

The data presented in this study are available when contact the corresponding author.

## Supplementary Materials

The following supporting information can be downloaded at: https://www.mdpi.com/article/10.3390/jcm12103548/s1. Table S1. PTs for all DIM cases reported in the FAERS database. Table S2. Identifying of statins and daptomycin with ATC codes. Table S3. Algorithm for disproportionate analyses.
