# Supplementary material for: Reporting of Drug-Induced Myopathies Associated with the Combination of Statins and Daptomycin: A Disproportionality Analysis Using the US Food and Drug Administration Adverse Event Reporting System
Source: J Clin Med. 2023 May 18;12(10):3548. doi: 10.3390/jcm12103548 (PMC10219483; doi:10.3390/jcm12103548)
Supplement: Supplementary file 1 [file jcm-12-03548-s001.zip › jcm-2365929-supplementary.pdf]

Reporting of Drug-induced Myopathies Associated with the Combination of Statins and Daptomycin: A Disproportionality Analysis Using the US Food and Drug Administration Adverse Event Reporting System

Chunyan Wei[1], Wanhong Yin[2], Jingyi Zhang[1], Huifang Shan[1], Yin Liu[1], Aidou Jiang[1], Bin Wu[1]

Supplementary Table S1. PTs for all DIM cases reported in the FAERS database.

NO.	PT[a] code	PT[a] name
1	10005672	Blood myoglobin increased
2	10028303	Muscle dissolution
3	10028320	Muscle necrosis
4	10028625	Myoglobin blood increased
5	10028627	Myoglobin urine increased
6	10028629	Myoglobinuria
7	10028631	Myoglobin urine present
8	10028636	Myonecrosis
9	10028641	Myopathy
10	10028642	Myopathy aggravated
11	10028646	Myopathy steroid
12	10028647	Myopathy steroid-induced
13	10028648	Myopathy toxic
14	10028649	Myopathy, unspecified
15	10032283	Other myopathies

16	10037078	Proximal myopathy
17	10037079	Proximal myopathy aggravated
18	10039020	Rhabdomyolysis
19	10042024	Steroid myopathy
20	10042753	Symptomatic inflammatory myopathy
21	10042754	Symptomatic inflammatory myopathy in diseases classified elsewhere
22	10044237	Toxic myopathy
23	10046646	Urine myoglobin increased
24	10058735	Myoglobin emia
25	10058781	Myoglobin emia
26	10059888	Myoglobin blood present
27	10066646	Acute myopathy
28	10072174	Axial myopathy
29	10074769	Necrotizing myositis
30	10074770	Necrotizing myositis
31	10081524	Thyrotoxic myopathy
32	10086278	Muscle infarction

[a]PT: prefer term

Supplementary Table S2. Identifying of statins and daptomycin with ATC codes.

NO.	Drug name	ATC[a] code	NO.	Drug name	ATC[a] code
1	Daptomycin	J01XX09	6	Fluvastatin	C10AA04

2	Simvastatin	C10AA01	7	Atorvastatin	C10AA05
3	Lovastatin	C10AA02	8	Cerivastatin	C10AA06
4	Pravastatin	C10AA03	9	Rosuvastatin	C10AA07
5	Pitavastatin	C10AA08			

[a]ACT: Anatomical Therapeutic Chemical

Supplementary Table S3. Algorithm for disproportionate analyses

Drugs	DIM[a] event cases	All other adverse event cases
Target drug	a	b
All other drugs	c	d

ROR[b] = $\frac{a/b}{c/d}$

95%CI[c] for ROR[a] = $e^{\ln(ROR)\pm 1.96\sqrt{(\frac{1}{a}+\frac{1}{b}+\frac{1}{c}+\frac{1}{d})}}$

IC[d] = $\log_2\frac{a(a+b+c+d)}{(a+b)(a+c)}$

E(IC[d]) = $\log_2\frac{(a+\gamma 11)(N+\alpha)(N+\beta)}{(N+\gamma)(a+b+\alpha 1)(a+c+\beta 1)}$

V(IC[d]) ≈ $(\frac{1}{\log 2})^2[\frac{N-a+\gamma-\gamma 11)}{(a+\gamma 11)(1+N+\gamma)}+\frac{N-a-b+\alpha-\alpha 1)}{(a+b+\alpha 1)(1+N+\alpha)}+\frac{N-a-c+\beta-\beta 1)}{(a+c+\beta 1)(1+N+\beta)}]$

$\gamma = \gamma_{11}\frac{(N+\alpha)(N+\beta)}{(a+b+\alpha 1)(a+c+\beta 1)}$

95%CI[c] for IC[d] = E(IC[d]) ± $1.96\sqrt{V(IC)}$

Where $\alpha=\alpha_1+\alpha_2$, $\beta=\beta_1+\beta_2$, N=a+b+c+d, and the value of α_1, α_2, β_1, β_2 and γ_{11} were defined as 1.

[a]DIM: drug-induced myopathies; [b]ROR: reporting odds ratio; [c]CI: confidence interval. [d]IC: information component.
